# The Barcelona Brain Health Initiative: Cohort description and first follow-up

**DOI:** 10.1371/journal.pone.0228754

**Published:** 2020-02-11

**Authors:** Gabriele Cattaneo, David Bartrés-Faz, Timothy P. Morris, Javier Solana Sánchez, Dídac Macià, Josep M. Tormos, Alvaro Pascual-Leone

**Affiliations:** 1 Institut Guttmann, Institut Universitari de Neurorehabilitació adscrit a la UAB, Badalona, Barcelona, Spain; 2 Universitat Autònoma de Barcelona, Bellaterra, Barcelona, Spain; 3 Departament de Medicina, Facultat de Medicina i Ciències de la Salut, Universitat de Barcelona, Barcelona, Spain; 4 Berenson-Allen Center for Noninvasive Brain Stimulation, Beth Israel Deaconess Medical Center, Harvard Medical School, Boston, MA, United States of America; 5 Hinda and Arthur Marcus Institute for Aging Research, Hebrew SeniorLife, Harvard Medical School, Boston, MA, United States of America; Nathan S Kline Institute, UNITED STATES

## Abstract

The Barcelona Brain Health Initiative is a longitudinal cohort study that began in 2017 and aims to understand and characterize the determinants of brain health maintenance in middle aged adults. A cohort of 4686 individuals between the ages of 40 and 65 years free from any neurological or psychiatric diseases was established, and we collected extensive demographic, socio-economic information along with measures of self-perceived health and lifestyles (general health, physical activity, cognitive activity, socialization, sleep, nutrition and vital plan). Here we report on the baseline characteristics of the participants, and the results of the one-year follow-up evaluation. Participants were mainly women, highly educated, and with better lifestyles compared with the general population. After one year 60% of participants completed the one-year follow-up, and these were older, with higher educational level and with better lifestyles in some domains. In the absence of any specific interventions to-date, these participants showed small improvements in physical activity and sleep, but decreased adherence to a Mediterranean diet. These changes were negatively associated with baseline scores, and poorer habits at baseline were predictive of an improvement in lifestyle domains. Of the 2353 participants who completed the one-year follow-up, 73 had been diagnosed with new neurological and neuropsychiatric diseases. Changes in vital plan at follow-up, as well as gender, sleep quality and sense of coherence at baseline were shown to be significant risk factors for the onset of these diagnoses. Notably, gender risk factor decreased in importance as we adjusted by sleep habits, suggesting its potential mediator effects. These findings stress the importance of healthy lifestyles in sustaining brain health, and illustrate the individual benefit that can be derived from participation in longitudinal observational studies. Modifiable lifestyles, specifically quality of sleep, may partially mediate the effect of other risk factors in the development of some neuropsychiatric conditions.

## Introduction

**Fig 1:**
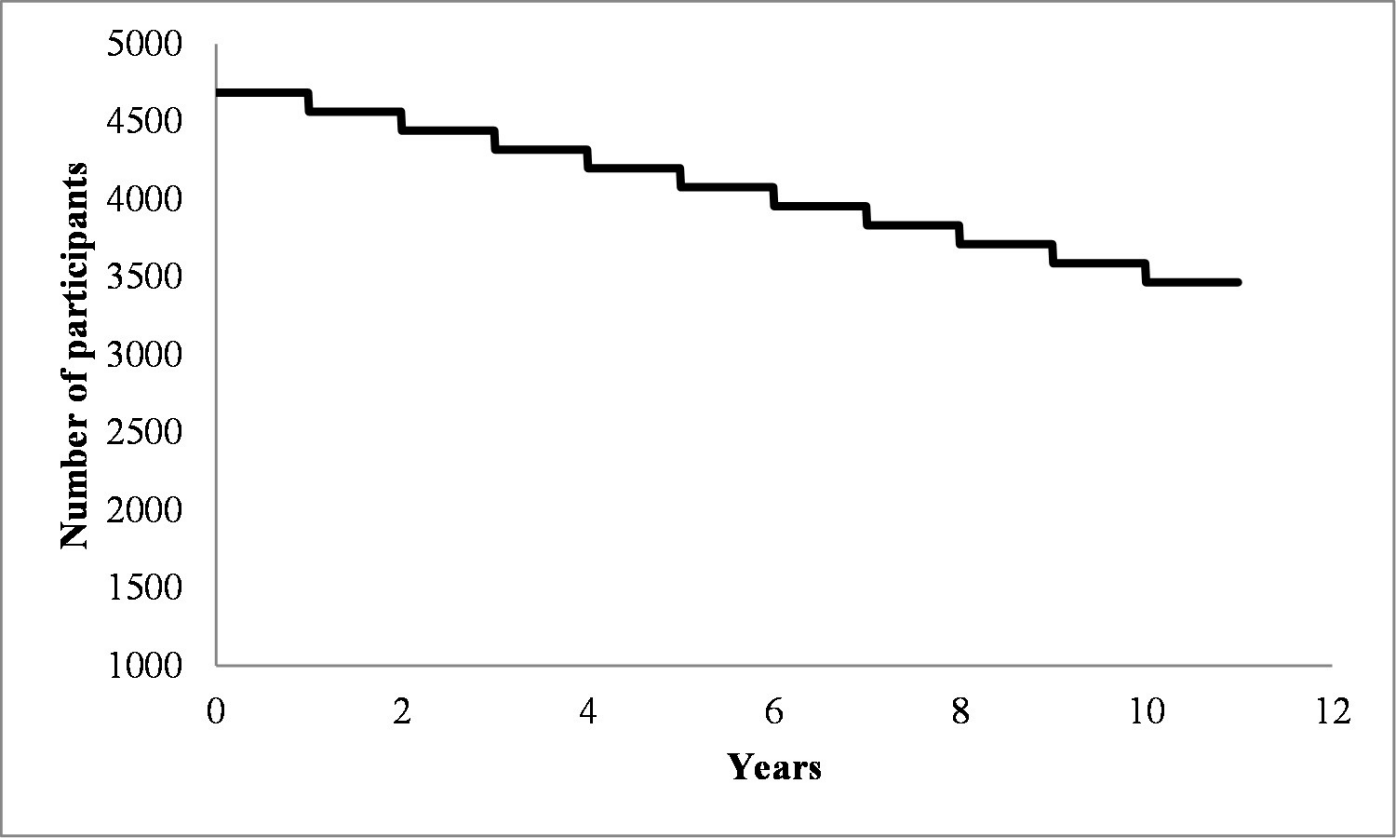
Graphical representation of the expected survival curve of the cohort for neurological and psychiatric diseases, based on epidemiological data [1–10].

The World Health Organization (WHO) makes it plain: “Neurological and mental disorders are the greatest threat to public health”, and it projects that they will be responsible for over half of the world-wide economic impact of disability by 2050. Unless we develop interventions to minimize the impact of emotional distress, learning disabilities, cognitive decline, brain and mental illnesses, society will face an unsurmountable crisis. Therefore, sustaining brain health and reducing the impact of neurological and psychiatric diseases is arguably the greatest challenge of biomedical research for the 21^st^ century. Indeed a systematic review of the literature reveals that if one were to identify a cohort of healthy individuals between 40 and 65 years of age, over the course of a decade, 26% would become diagnosed with neurological or psychiatric diseases [1](see Fig 1).

Among the younger group, particularly women might be expected to be diagnosed with depression [11,12], while among the older group, mild cognitive impairment and dementia would be predicted to be the most common diagnoses [2,4,6].

A growing number of studies have investigated the mechanisms contributing to the preservation of brain structure and function, cognitive performance, and mental wellbeing, as well as the role of compensation in the face of injury or disease [13,14]. There is increasing evidence that some dietary patterns, leisure activity, cognitive stimulating activity, physical activity, as well as other social and psychological activities can promote brain health maintenance and are associated, for example, with lower risk of dementia (see [15] for a systematic review). Livingstone and collaborators suggested that even beyond the biological or genetic predisposition (e.g. Apoe4 allele), dementia could be prevented in up to 35% of the cases through improved control of medical conditions such as hypertension, obesity and diabetes, and attention to modifiable risk factors such as education, smoking, physical activity and socialization. Recent interventional studies support this view and suggest that multi-domain lifestyles interventions may improve mental and cognitive functioning, and be particularly effective in people at risk [16]. Thus, modifiable and non-modifiable factors may interact and ultimately either increase or decrease an individual’s risk to develop dementia and other neurological or psychiatric conditions [15, 17–19].

The Barcelona Brain Health Initiative (BBHI) is a longitudinal cohort study started in 2017 with the aim to understand and characterize biological, behavioral and environmental markers, and their interactions, related with brain health maintenance in middle to old age [1]. In the first phase of the study, participants completed questionnaires that collected information about socio-demographic, socio-economic, and lifestyles relevant to brain health. We hypothesized that participation in the study might increase awareness of the importance of healthy lifestyles, improving adherence. Here we 1) report baseline characteristics of the cohort and analyze the changes on lifestyles measures after one year of participation in the study, and 2) identify participants who developed new neurological and psychiatric diagnoses and identify risk factors that contributed to them.

## Material and methods

### Participants

BBHI study participants are community-dwelling individuals, between 40 and 65 years, free from any self-reported neurological or psychiatric diagnosis at the time of recruitment [1].

Participants were recruited by an intensive dissemination and communication campaign made through different media (TV, radio, newspapers) as well as the most common social media platforms (Facebook, Twitter, etc.). The engagement of well-known opinion leaders from the sports, journalism or acting world, who recorded short videos describing the study and thus served as ambassadors for the initiative, helped increase the visibility and contributed to the recruitment.

People interested in the study enrolled online through the website of the project (https://bbhi.cat) by filling out an initial contact form. Upon confirmation of their email, they gave on-line informed consent to the study which had been approved by the Institutional Review Board of the Institute Guttmann neurorehabilitation hospital. Thereafter participants created their personal profiles and completed an initial on-line questionnaire.

A total of 4,686 participants, mean age = 53.2 years fulfilled inclusion criteria and completed the initial online questionnaire.

### Baseline evaluation

Following the initial questionnaire (see Table 1), which had been validated as reported in Cattaneo et al. 2018, participants were asked to complete several additional validated questionnaires on-line (between July and December 2017) to further explore their self-perceived quality of life and health, their engagement in cognitive activities, physical activities and socialization, their habits in nutrition and sleep, their psychological well-being and vital plan, as well as other information (see Table 2).

**Table 1:** Initial questionnaire administered at enrollment.

**Self-perceived General Health**	PROMIS [22]
**Self-perceived Mental Health**	PHQ-4 [23]
**Cognitive Complaints**	Neuro-Qol [24]
**Physical activity**	Physical Activity Questionnaire (PAQ)
**Sleep**	Jenkins Sleep Evaluation Questionnaire (JSEQ, [25])
**Nutrition**	Mediterranean Diet Adherence screener (MeDas; [26]
**Vital Plan**	Personal growth and Purpose in life subscales of Ryff’s scale of Psychological well-being [27]

**Table 2:** Subsequent questionnaires characterizing various lifestyle domains.

**Cognitive Reserve**	Cognitive Reserve Questionnaire (CRQ, [28])
**Physical Activity**	Godin Leisure Time Physical Activity Questionnaire (GLTPAQ, [29])
**Vital Plan**	The Engaged Living Scale (ELS;[30])
	Subjective Self Health Horizon Questionnaire (SHH-Q,[31])
	Sense of Coherence scale (SOC; [32])
	Ryff scale of Psychological well-being [27]
**Socialization**	Lubben Social Network Scale (LSNS; [33])
**Sleep**	Pittsburg Sleep Quality index (PSQI; [34])
**Emotional Health**	Depression Anxiety and Stress Scale (DASS; [35])
**Quality of Life**	The World Health Organization Quality of Life Assessment (WHOQOL-AGE, [36])
**Hand dominance**	Edinburgh Handedness Inventory (EHI, [37])
**Bilingualism**	Questionnaire of bilingualism and multilingualism (BIL, [38])

To evaluate Vital plan we consider in particular three dimensions that composed the construct of “Meaning in Life”: sense of coherence, purpose in life and engagement with life [20]. The first is a cognitive component, consisting of the ability to make sense of our experiences, the second is motivational and is about having a goal in life, while the third is an affective component related with satisfaction with life (see [21]).

### One year follow-up evaluation

In November 2018 we conducted a first follow-up evaluation, re-administering the initial questionnaires, with some components expanded (see Table 3).

**Table 3:** Questionnaires administered for the 1-year follow-up.

**Self-perceived General Health**	PROMIS [22]
**Self-perceived Mental Health**	PHQ-4 [23]
**Cognitive Complaints**	Neuro-Qol [24]
**Physical activity**	PAQ, International Physical Activity Questionnaire (IPAQ,[39])
**Cognitive reserve**	CRQ [28]
**Sleep**	JSEQ [25], STOP-Bang questionnaire [40], Single question RBD [41]
**Socialization**	LSNS [33], UCLA Loneliness scale [42]
**Nutrition**	MeDas [26]
**Vital Plan**	Personal growth and Purpose in life subscales of Ryff’s scale of Psychological well-being [27], Meaning in life questionnaire [43]
**Alcohol Consumption**	AUDIT [44]
**Smoking**	Pack-year index

### Feedback provided to participants

An important methodological aspect of the BBHI is that after completing every questionnaire, participants receive a graphic feedback of their responses as they relate to each one of the areas defined as “brain health pillars”: cognitive function, physical activity, sleep, nutrition, socialization and purpose in life [1]. Moreover we periodically publish in our web page tips and news related with brain health and healthy lifestyles in these domains.

**Fig 2:**
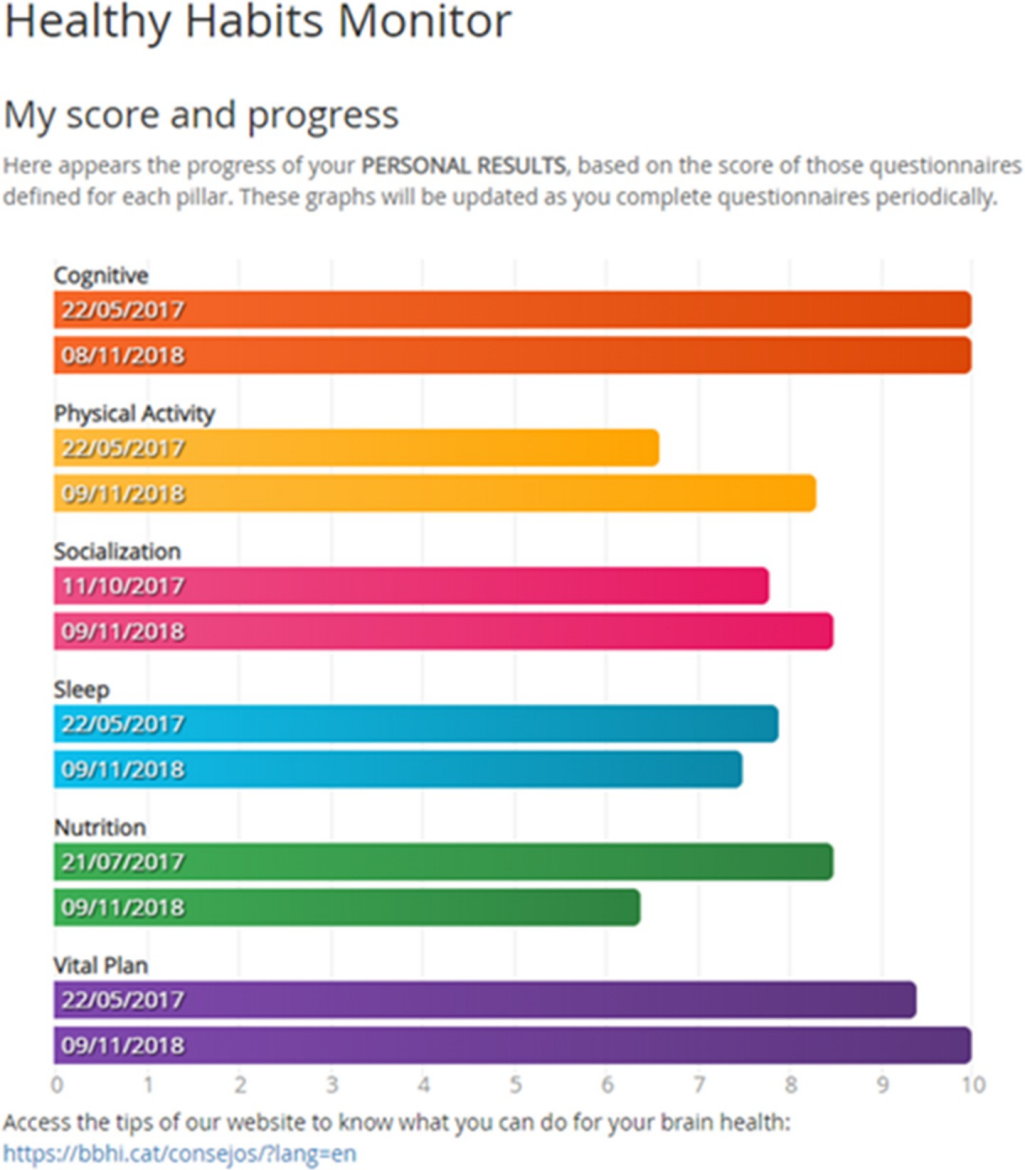
Position of participants respect to other similar participants.

At the follow-up evaluation participants were given feedback providing the same graphical representation of 6 circle graphs, one for each brain health pillar, but representing now the percentile they occupy when compared with the rest of the BBHI participants of the same gender and age bracket (± 5 years; Fig 2).

**Fig 3:**
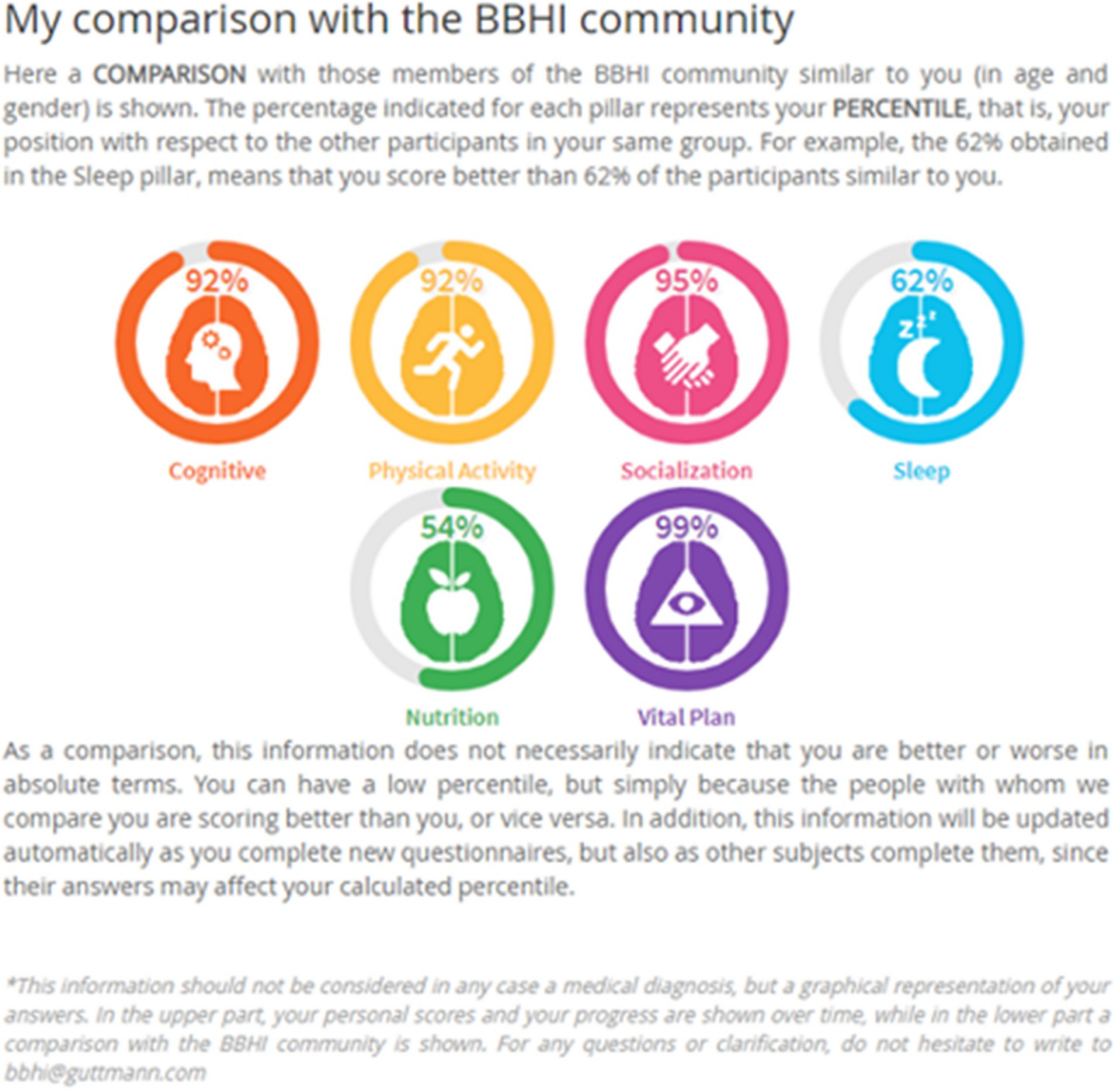
An example of a participant’s change in lifestyle adherence over time: comparison of responses in the first wave of questionnaires and at the follow-up.

In addition, participants are provided a second form of feedback of their changes over time (Fig 3), where each bar represents the translated score from 0 to 10 for each pillar, so users can easily compare their evolution (inside each bar the date when users responded is shown).

### Data and statistical analysis

Data analysis was performed in consecutive steps. First, a descriptive analysis of the baseline characteristics of all cohort participants was completed. We also explored the association between baseline lifestyles and other medical conditions we ran Spearman correlation and binomial logistic regression analyses.

To test if there were any differences in baseline scores between those participants who did or did not complete the one-year follow up we used two-sample t test and Chi-squared statistics. Third, we assessed possible changes between baseline and follow-up using paired t tests.

Next, to explore whether baseline characteristics were predictive of changes over time for each of our variables of interest we regressed its change over time (one-year follow up score—baseline score) on its baseline score. To correct for “regression to the mean”, due to intrinsic noisy nature of the data collecting instruments, we re-ran linear regressions using the changes (subtraction between follow-up and baseline) as dependent variables and the mean between first and second questionnaires’ scores as predictors (after after checking for variance homogeneity; [45,46].

Finally, to assess the associations between lifestyle and sociodemographic variables and the development of a new neurological of psychiatric diagnosis multiple binomial logistic regression was performed. We report the relative risk as beta coefficients with 95% confidence intervals. We additionally performed mediation analysis to determine whether modifiable risk factors mediated the putative effects of non-modifiable factors on a new diagnosis applying Hayes’s mediation model (model 4 [47]) using the PROCESS tool for SPSS [48]. Statistical analyses were performed using SPSS version 20.0 (Statistical Package for Social Sciences, Chicago, IL, USA).

## Results

### Baseline characterization of the cohort

The socio-demographic characteristics of the BBHI cohort are summarized in Table 4. As noted in [1], women (biological sex) are over-represented (66.8% v.s 50.9%), and participants have a higher educational level than the general population of Catalonia (71,7% v.s 41.2%). People living alone are underrepresented (14.0% v.s 23.3%) while other living situations are in line with those of the general population. Occupational status is consistent with the full population of people of the same age in Catalonia (data from the Statistical Institute of Catalonia; www.idescat.cat), as well as living town situation.

**Table 4:** Socio demographic characteristics of participants broken for biological sex.

	Men		Women		Total	
	***Mean (SD)***		***Mean (SD)***		***Mean (SD)***	
**Age (years)**	*53*.*8 (7*.*0)*		*53*.*0 (6*.*7)*		*53*.*2 (6*.*8)*	
	**Men**		**Women**		**Total**	
	***N***	***%***	***N***	***%***	***N***	***%***
**Participants**	*1555*	*33*,*2*	*3131*	*66*,*8*	*4686*	*100*,*0*
**Marital status**	*** ***		* *	* *	* *	* *
Married	*1051*	*67*,*6*	*1813*	*57*,*9*	*2864*	*61*,*1*
Separated/Divorced	*229*	*14*,*7*	*592*	*18*,*9*	*821*	*17*,*5*
Widowed	*15*	*1*,*0*	*84*	*2*,*7*	*99*	*2*,*1*
Never married	*260*	*16*,*7*	*642*	*20*,*5*	*902*	*19*,*2*
**Living status**	*** ***	* *	*** ***	*** ***	*** ***	*** ***
Alone	*200*	*12*,*9*	*454*	*14*,*5*	*654*	*14*,*0*
With partner without children	*363*	*23*,*3*	*630*	*20*,*1*	*993*	*21*,*2*
With partner and children	*849*	*54*,*6*	*1513*	*48*,*3*	*2362*	*50*,*4*
With children without partner	*64*	*4*,*1*	*386*	*12*,*3*	*450*	*9*,*6*
With parents	*29*	*1*,*9*	*45*	*1*,*4*	*74*	*1*,*6*
Others	*50*	*3*,*2*	*103*	*3*,*3*	*153*	*3*,*3*
**Educational status**	*** ***	*** ***	* *	* *	* *	* *
No studies	*1*	*0*,*1*	*0*	*0*,*0*	*1*	*0*,*0*
Primary	*73*	*4*,*7*	*128*	*4*,*1*	*201*	*4*,*3*
Secondary	*437*	*28*,*1*	*689*	*22*,*0*	*1126*	*24*,*0*
Higher	*1044*	*67*,*1*	*2314*	*73*,*9*	*3358*	*71*,*7*
**Occupational status**	*** ***	* *	*** ***	*** ***	*** ***	*** ***
Retired	*145*	*9*,*3*	*202*	*6*,*5*	*347*	*7*,*4*
Unemployed	*92*	*5*,*9*	*297*	*9*,*5*	*389*	*8*,*3*
Part-time	*97*	*6*,*2*	*360*	*11*,*5*	*457*	*9*,*8*
Full-time	*1221*	*78*,*5*	*2272*	*72*,*6*	*3493*	*74*,*5*
**Type of work**	*** ***	*** ***	*** ***	*** ***	*** ***	*** ***
Unqualified manual	*50*	*3*,*2*	*92*	*2*,*9*	*142*	*3*,*0*
Qualified manual	*370*	*23*,*8*	*795*	*25*,*4*	*1165*	*24*,*9*
Professional	*369*	*23*,*7*	*1015*	*32*,*4*	*1384*	*29*,*5*
Manager	*529*	*34*,*0*	*730*	*23*,*3*	*1259*	*26*,*9*
**Household monthly income**	*** ***	*** ***	*** ***	*** ***	*** ***	*** ***
Less than 1,000€	*52*	*3*,*3*	*132*	*4*,*2*	*184*	*3*,*9*
Between 1,000 and 2,000 €	*263*	*16*,*9*	*766*	*24*,*5*	*1029*	*22*,*0*
Between 2,000 and 5,000 €	*938*	*60*,*3*	*1782*	*56*,*9*	*2720*	*58*,*0*
More than 5,000 €	*302*	*19*,*4*	*451*	*14*,*4*	*753*	*16*,*1*
**Living town**	*** ***	*** ***	*** ***	*** ***	*** ***	*** ***
Small town (less than 2,000 inhabitants)	*66*	*4*,*2*	*158*	*5*,*0*	*224*	*4*,*8*
Town (less than 20,000 inhabitants)	*346*	*22*,*3*	*665*	*21*,*2*	*1011*	*21*,*6*
City (more than 20,000 inhabitants)	*572*	*36*,*8*	*1032*	*33*,*0*	*1604*	*34*,*2*
Big city (more than 500,000 inhabitants)	*571*	*36*,*7*	*1276*	*40*,*8*	*1847*	*39*,*4*

### Baseline lifestyles

Participants performed on average 2 hour of moderate to vigorous aerobic activity per week and only 5.3% of participants showed low adherence to Mediterranean diet; the whole cohort showed a medium to high adherence (mean ± standard deviation = 8.8 ± 2.0) to it. Scores on the Jenkins Sleep Evaluation Questionnaire (8.5 ± 3.8) and Pittsburg Sleep Quality Index (5.7 ± 2.9) indicate a slight trend to poor sleep quality [34]. Socialization instead was quite high (36.1 ± 9.0) and far from the “clinical” risky cut-off of 20 [33]. When we looked at the distribution of scores in Sense of coherence (66.2 ± 11.3), Engagement with life (61.0 ± 9.2) and Ryff psychological well-being (151.4 ± 19.6) only 3-to-3.5% of participants showed scores below mean– 2 standard deviations.

### Baseline lifestyles and medical conditions

**Fig 4:**
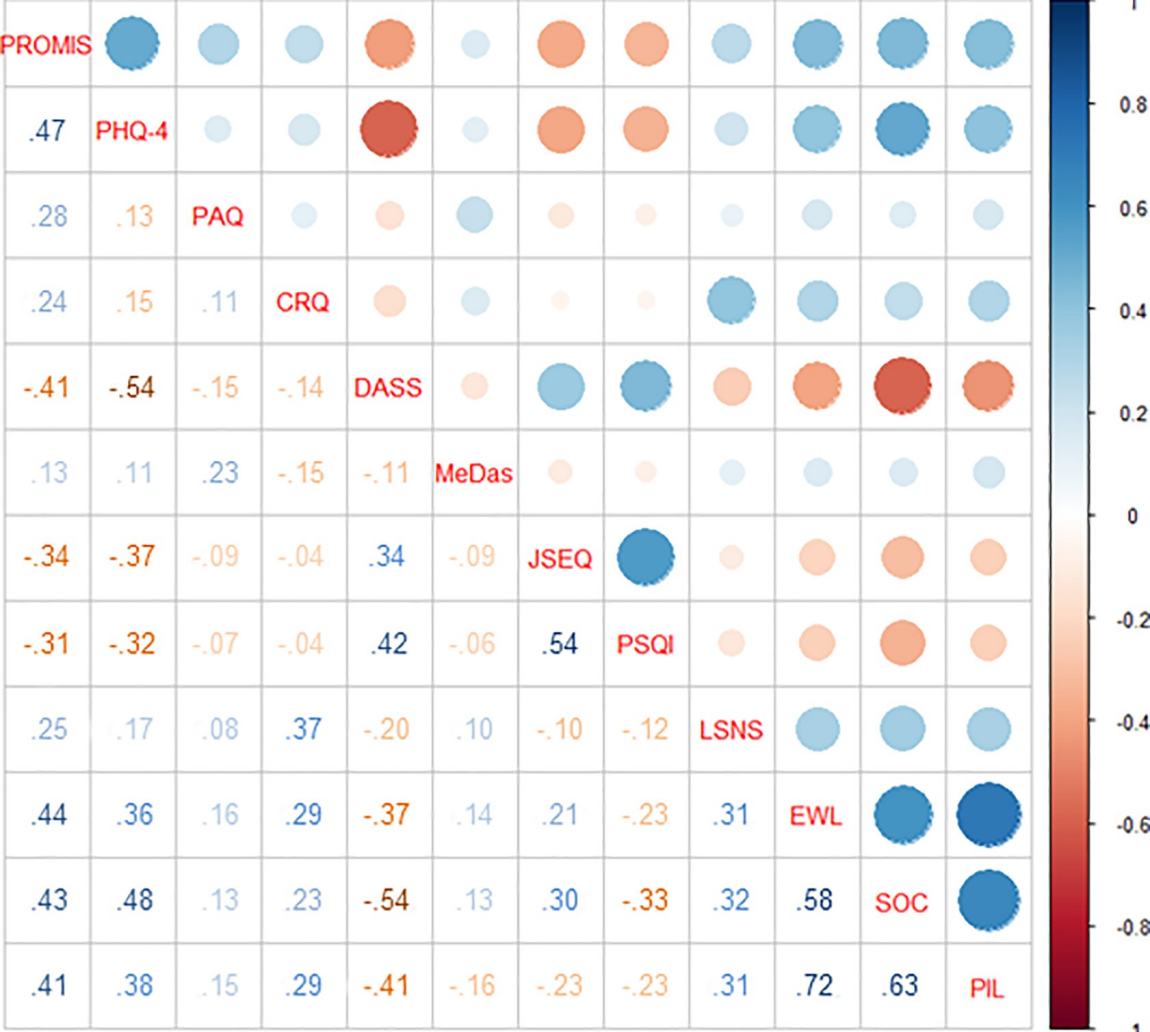
Graphical representations of the correlation’s strength between different lifestyles at baseline. Numbers in the table are ρ coefficients.

When we explored the relation between baseline lifestyles we found significant associations between all of them (all p values ≤0.01), even if physical activity and adherence to Mediterranean were weekly associated to other lifestyles (see Fig 4).

Moreover we found that less physical activity and lower sleep quality, measured both with the Jenkins Sleep Evaluation Questionnaire and Pittsburg Sleep Quality Index, were associated with the presence of other medical conditions than neurological and psychiatric ones (see Table 5).

**Table 5:** Multiple logistic binomial regression using any medical condition (not neurological or psychiatric) as dependent variable, lifestyles at baseline as independent variables, and gender and age as covariates. Reported values are standardized beta coefficients.

**Variable**	**B (C.I. 95%)**	**p**	** **
Physical activity**	**0.95 (0.93–0.97)**	**<0.001**	*
Sleep quality (JSEQ)**	**1.04 (1.02–1.07)**	**<0.001**	*
Sleep quality (PSQI)**	**1.06 (1.03–1.09)**	**<0.001**	*
Mediterranean diet adherence**	**0.99 (0.96–1.03)**		
Engagement with Life**	**1 (0.99–1.01)**		
Sense of coherence**	**1 (0.99–1.01)**		
Purpose in Life**	**1 (0.98–1.02)**		
Cognitive reserve**	**1 (0.98–1.02)**		
Emotional health**	**1 (0.98–1.02)**		
Socialization**	**1.01 (1.00–1.01)**		
**Age**	**1.11 (1.10–1.12)**	**<0.001**	*
**Gender**	**0.6 (0.51–0.70)**	**<0.001**	*

* C.I. which do not include 1 are considered statistically significant

** Data were recollected with questionnaires reported in Table 2.

The medical conditions we consider for the analysis were: Hypertension, hearth problems or heart attack, hypercholesterolemia, diabetes, chronic pain, hepatitis, cirrhosis, pancreatic problems, sleep apnea, arthritis, chronic nephritis or cancer.

### 1-year follow-up responders’ profile

Of the 4686 participants who completed the baseline set of questionnaires, 2357 completed the one-year follow-up assessment (50%).

Participants who complete the one-year follow up questionnaire were older (53.8 ± 6.7; t = -5.70, p<0.001, d = 0.17) compared with those who did not (mean age ± standard deviation = 52.7 ± 6.8) and more educated (t = 4.34, p<0.001, d = 0.13), while there were no differences in the gender distribution (X^2^ = 0.01; p = 0.951).

**Fig 5:**
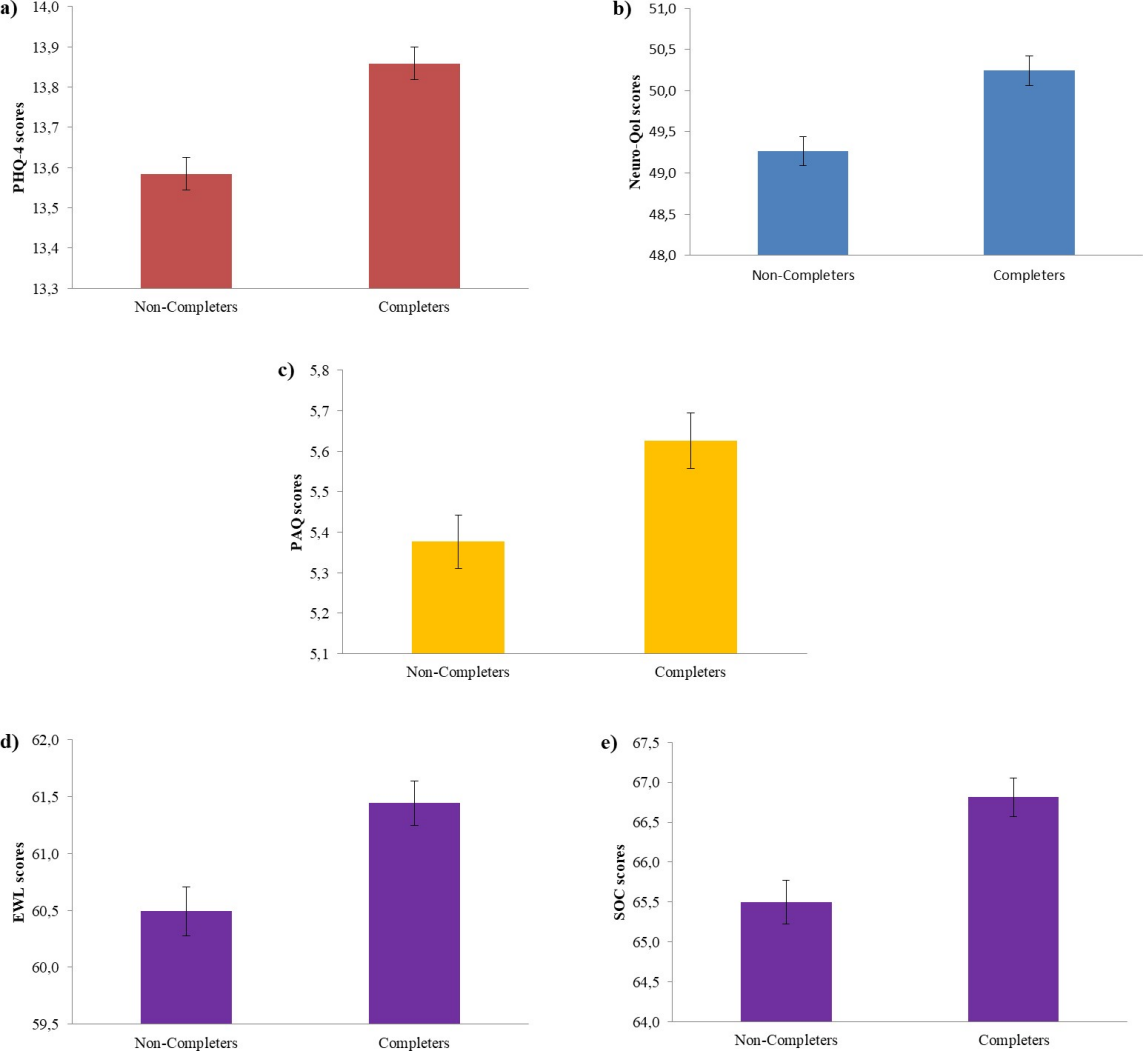
Comparison of baseline lifestyles scores (y axis) of participants who did and did not complete the follow-up (x axis) in a) Emotional health, b) Cognitive health, d) Physical activity, e) Engagement with life, and e) Sense of coherence.

Completers reported better self-perceived emotional (t = 4.78, p<0.001, d = 0.14) and cognitive health (t = 3.86, p<0.001, d = 0.11) at baseline. Furthermore, individuals completing the follow-up evaluation reported at baseline more physical activity (t = 2.61, p = 0.009, d = 0.08), engagement with life (t = 3.27, p = 0.001, d’ = 0.10) and higher sense of coherence scale scores (t = -3.63, p<0.001, d = 0.12; see Fig 5).

### 1-year follow up

**Fig 6:**
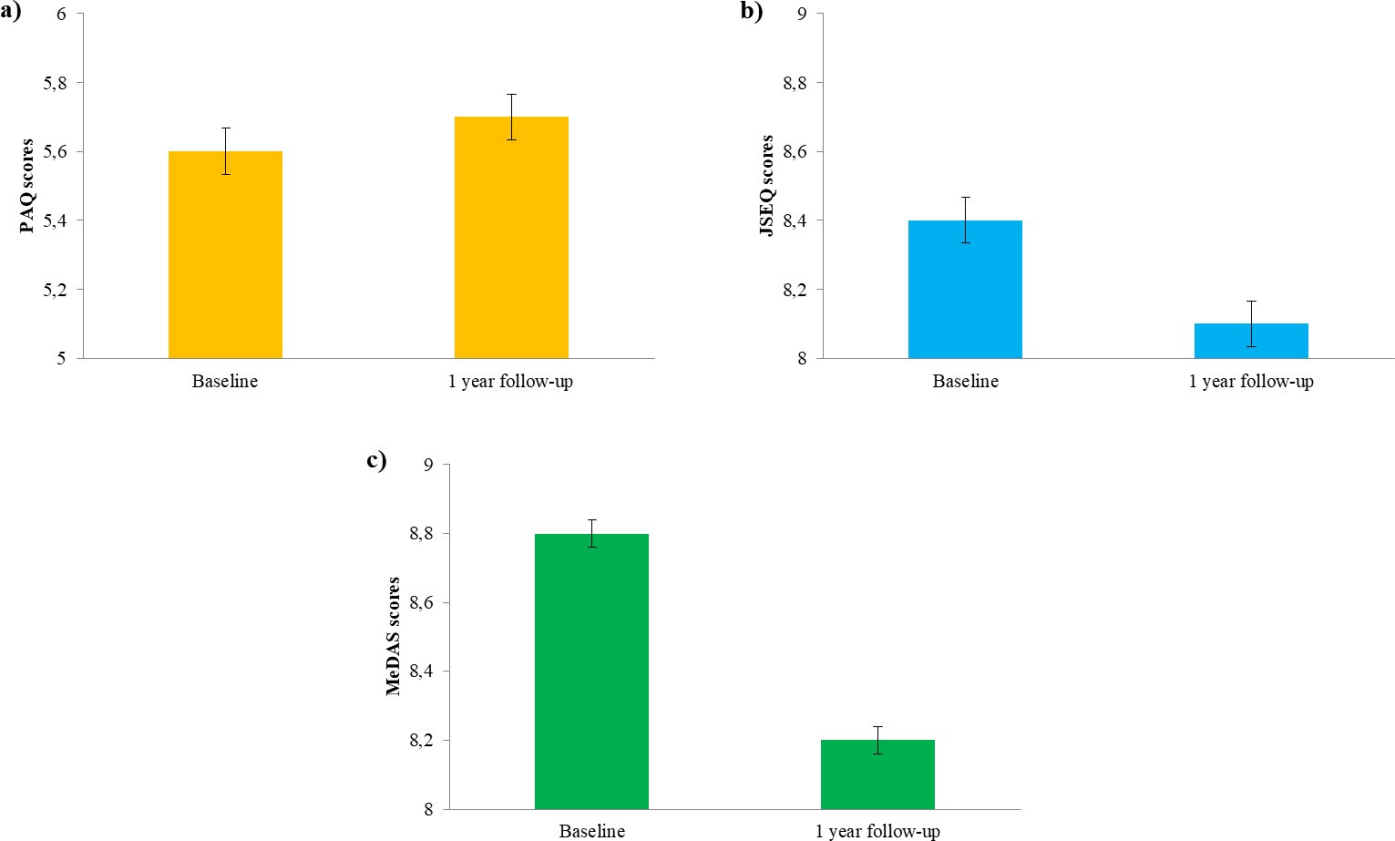
Comparison of participant’s score at the different lifestyles surveys at the baseline and after one year in a) Physical activity, b) Sleep complaints (scale is inverted), c) Adherence to Mediterranean diet. Error bars represent standard errors.

For the participants who completed the one-year follow-up assessment, self-reported lifestyles showed improvements in physical activity (t = -2.27, p = 0.023, d’ = 0.07) and quality of sleep (t = 4.37, p<0.001, d’ = 0.09) while nutrition worsened in the follow-up as compared with baseline (t = 17.14, p<0.001, d’ = 0.30; see Fig 6). However, some of these statistical significant differences were very small in effect size and thus should be interpreted carefully. No change was seen in any of the other questionnaires.

**Fig 7:**
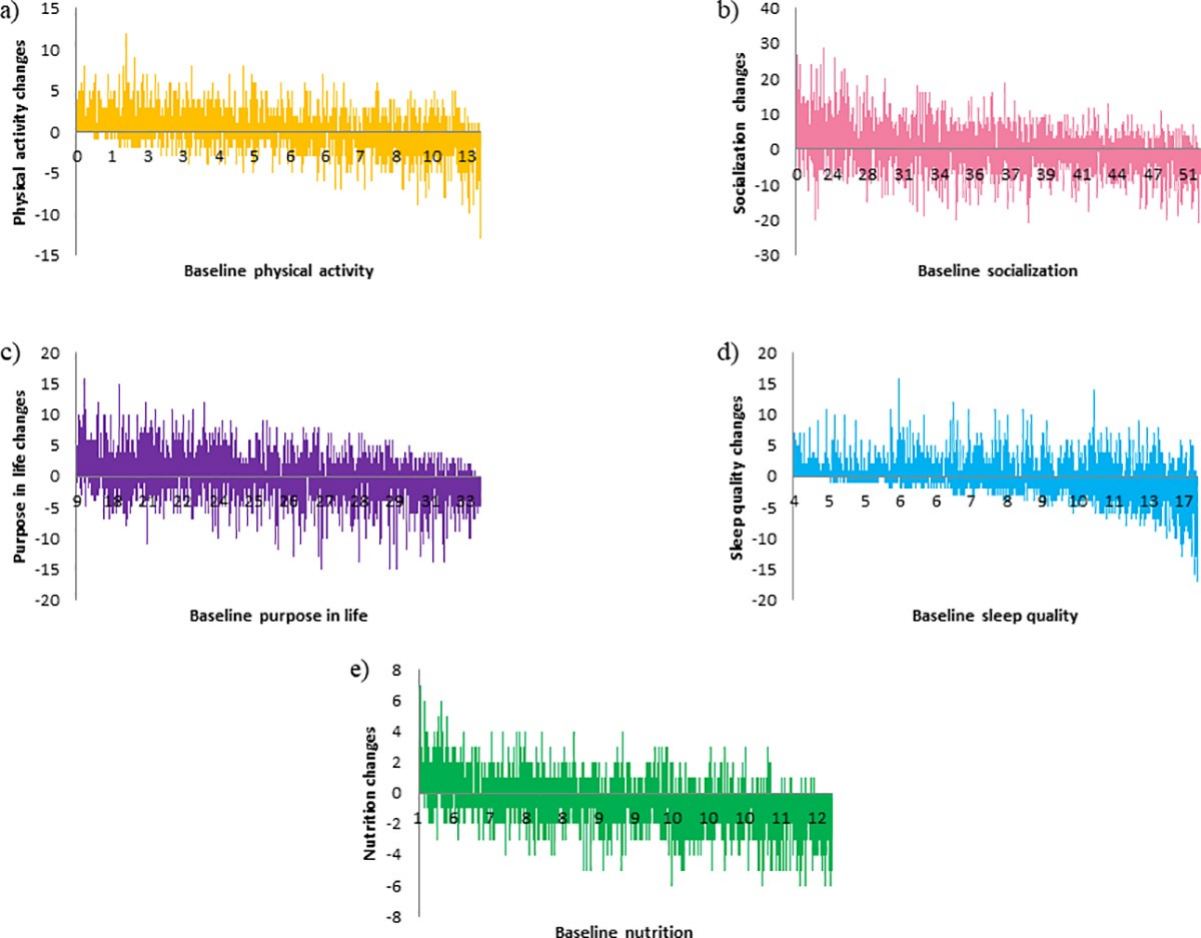
In the x axis of the waterfall plot baseline scores are reported and in the y axis changes between baseline and the follow-up are reported in the different domains: a) physical activity, b) socialization, c) purpose in life, d) sleep and e) nutrition.

We observed that individuals with lower scores at baseline across the different questionnaires, tended to exhibit better lifestyle habits (i.e. higher scores) at follow-up. This tendency was however inverted for those with higher ratings at baseline (see Table 6 below). Waterfall plots in Fig 7 represent follow–up changes (y axis) in function of baseline scores (x axis). Bars represent the larger positive and negative change for every baseline score of participants.

**Table 6:** Linear regressions between habit changes and baseline scores. Number in table are standardized beta coefficient.

Physical activity	Socialization	Purpose in Life	Sleep	Nutrition
-0.40**	-0.34**	-0.32**	-0.44**	-0.47**

** p<0.001

When we tried to correct for regression to the mean we confirmed that in the domains of nutrition and purpose in life people with worse habits at baseline were those who made greater improvements at the one year follow-up, and the opposite was true for those with better habits at baseline (see Table 7 below).

**Table 7:** Linear regressions between habit changes and mean scores (first and second questionnaires). Numbers in table are standardized beta coefficient.

Physical activity	Socialization	Purpose in Life	Sleep	Nutrition
-0.02	-0.02	-0.08**	-0.02	-0.06*

*p = 0.003

** p<0.001

### New neurological and psychiatric diagnoses at one year follow-up

At the one-year follow up assessment we asked participants for new medical conditions that had been diagnosed by their physicians during the last year, since their enrollment in the project and the completion of the initial survey.

Of the 2353 people who responded to the follow-up survey 73 people (3.1%) reported having been given a formal new diagnosis of neurological or psychiatric condition by their physicians (Table 8). As expected for our cohort characteristics, and in line with current epidemiological literature (Ferrari et al., 2013), the most frequent new diagnosis was major depression (76.7%). Also noteworthy are mild cognitive impairment or dementia (8.2%), and Parkinson’s disease (5.5%).

**Table 8:** Frequency of participant’s new medical conditions that represent an endpoint for the study.

Diagnosis	N
**Depression**	56
**Alzheimer's disease or Mild cognitive impairment**	6
**Parkinson's disease**	4
**Stroke**	2
**Multiple Sclerosis**	2
**Epilepsy**	2
**Lateral Amyotrophic Sclerosis**	1
**Schizophrenia/Psychosis**	0
**TOT**	73

Gender (Specifically woman), poor sleep quality and lower sense of coherence at baseline carried significant relative risks for the development of depression (Table 9). In addition, Table 10 shows the relative risk of the change in modifiable lifestyle factors and the emergence of depression. A change in one’s vital plan was significantly associated with a reduced risk of developing a new diagnosis.

**Table 9:** Multiple logistic binomial regression using new diagnosis of depression as dependent variable and baseline characteristics of participants as independent variables. Reported values are standardized beta coefficients.

Variable	B (C.I. 95%)	p	
**Age**	1.0 (0.9–1.1)		
**Gender (Women)**	4.8 (1.8–12.4)	0.001	*
**Education**	1.2 (0.7–2.0)		
**Employment**	1.1 (0.8–1.6)		
**Living in a big town**	1.0 (0.7–1.4)		
**Family income**	0.8 (0.51.2)		
Emotional complaints**	0.9 (0.8–1.0)		
Cognitive complaints**	1.0 (0.9–1.1)		
Physical activity**	1.0 (0.9–1.1)		
Socialization**	1.0 (0.9–1.1)		
Sleep Quality**	1.1 (1.0–1.2)	0.006	*
Mediterranean diet adherence**	1.1 (0.9–1.3)		
Engagement with life**	1.0 (0.9–1.1)		
Sense of Coherence**	0.9 (0.9–1.0)	0.003	*
Purpose in Life**	1.0 (0.9–1.1)		

* C.I. which do not include 1 are considered statistically significant

** Data were recollected in the first questionnaire and by PHQ-4 [23] NeuroQoL [24], LSNS [33], JSEQ [25], MeDAS [26], EWL [30], SOC [32], PIL [27].

**Table 10:** Multiple logistic binomial regression using new diagnosis of depression as dependent variable, lifestyles changes at follow-up as independent variables, and gender and age as covariates. Reported values are standardized beta coefficients.

Variable	B (C.I. 95%)	p	
**Physical activity change**	1.0 (0.9–1.1)		
**Socialization changes**	1.0 (0.9–1.1)		
**Purpose in Life changes**	0.9 (0.9–1.0)	0.05	*
**Sleep quality changes**	1.0 (0.9–1.1)		
**Nutrition changes**	0.9 (0.7–1.0)		

* p≤0.05

### The mediator effect of modifiable lifestyles over non-modifiable risk factors

To investigate the relation between modifiable and non-modifiable risk factor on new diagnosis of depression we applied mediation models. A preliminary regression and found that gender was not related with sense of coherence (p = 0.95), and so no mediation model was performed on these variables.

**Fig 8:**
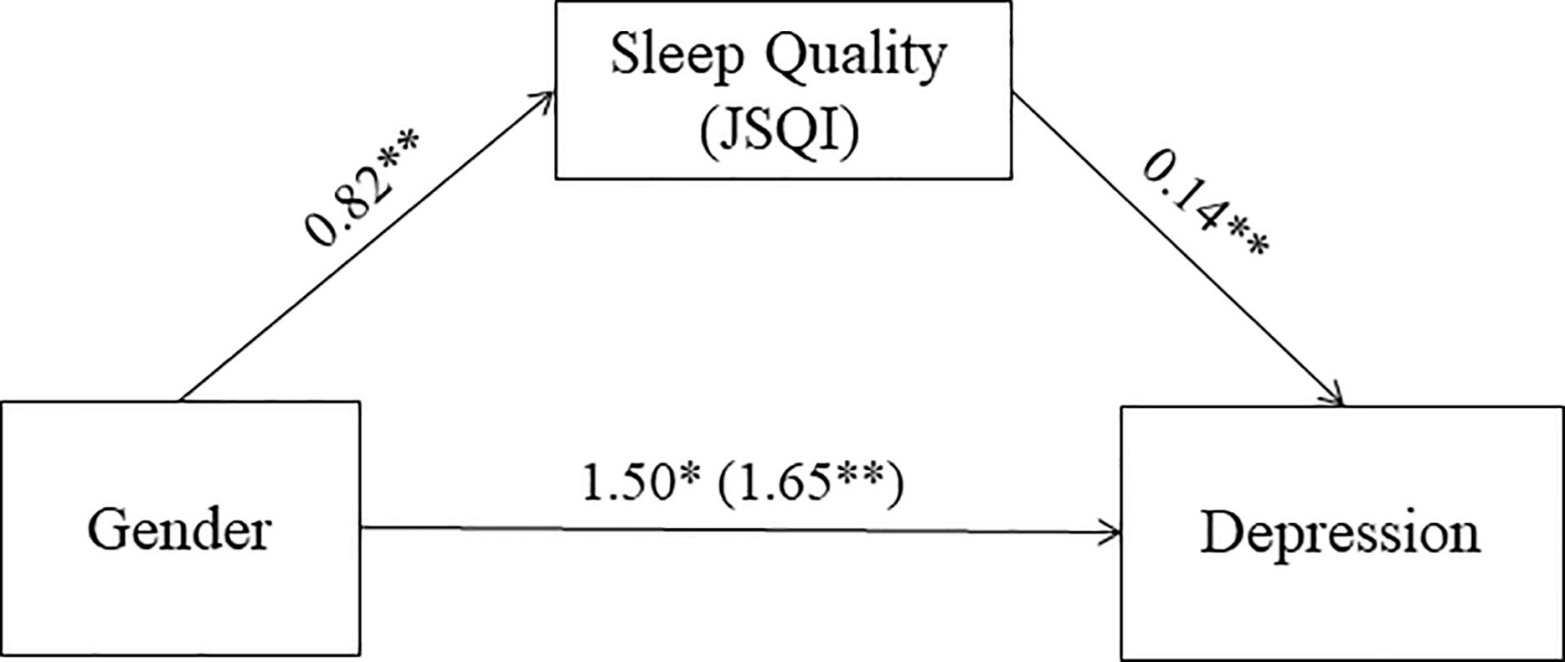
JSEQ score as mediator of the association between gender and new diagnosis of depression at the one-year follow-up. Values are B unstandardized coefficients (*p = 0.001; **p < 0.001); values within parentheses represent total relationship between Gender and new diagnosis when JSEQ are not take into consideration.

A second analysis revealed that sleep quality significantly and partially mediated the association between gender and the development of new diagnosis of depression after one year (95% CI, 0.06–0.18; Fig 8).

## Discussion

Over the course of the one-year follow-up we found that older participants, with more education and better lifestyle habits were more likely to continue to engage with the study. Of those who completed the one-year follow-up, physical activity and quality of sleep improved, whereas adherence to Mediterranean diet decreased. Those with lower baseline scores improved their habits over time, while those with healthier habits at the beginning tended to decrease their adherence to them. Such an effect of participation in observational longitudinal study on modifiable lifestyles and cognitive and mental wellbeing are important to consider in assessing the impact of possible targeted interventions.

When we looked at the relation between new diagnosis, baseline characteristics, and change over one year of study participation, we found that a well-defined vital plan appears to offer some protection for a new diagnosis of depression, while female gender, poor sleep quality and low sense of coherence predicted the onset of a new diagnosis. Of these, sleep appears to mediate the relationship between non-modifiable factors, such as gender, and new diagnoses of depression.

### Baseline characteristics of the cohort

Participants were not representative of the general population for socio-demographic factors and lifestyles, probably due to the study methodology (on-line recruitment). As in other cohort studies women are over-represented and participants have higher educational level than the general population [49]. Also the on-line administration of questionnaires may introduced a bias in cohort representativeness and well as possible confounders/limitations.

Participants showed at baseline, on average, a medium to high adherence to healthy lifestyles. Their 2 hour of moderate to vigorous aerobic activity per week is in line with the 150 minutes of moderate intensity exercise or 75 minutes of vigorous exercise per week recommended by the World Health Organization and the American College of Sports Medicine [50, 51]. Adherence to Mediterranean diet was in general quite high and the proportion of people with low adherence to it was less than half of what found in the Catalan population (13.8%; [52]). Socialization levels (score 36.1) were sufficiently high suggesting that in the total cohort, participants were far from “at risk” for social inactivity or isolation (score <20; [33]). Interestingly, sleep quality is the worst reported lifestyles and tended to be poorer than what is recommended (score <5 at PSQI 4]. For meaning in life, it is difficult to evaluate the significance of the level of Sense of coherence, Engagement with life and Ryff’s psychological well-being scores of participants quantitatively, considering the absence of precise recommendations in literature.

This medium to high adherence to some health related lifestyles of our participants reflect the general tendency culturally related of Catalan/Spanish population. For this reason Barcelona was an ideal location to study the effect of good lifestyle habits on the maintenance of brain health given a lot of the lifestyle pillars we study are inherent in the culture (diet, exercise, socialization for example).

When we explored the relation among lifestyles in the different domains we found a strong relation between all of them. People with healthy lifestyles in one domain showed the same degree of healthy lifestyles also in all the others domains.

Finally, when we looked at the relation between lifestyles and medical conditions other than neurological and psychiatric ones we found a clear association between and higher level of physical activity and good quality of sleep with less prevalence of medical diagnosis.

### Retention challenges

Retention at the one-year follow-up is clearly a limitation of the study, and is an important issue to explore. About 50% of the participants who enrolled failed to complete the one-year follow-up survey. Such an attrition rate is not far from previous on-line longitudinal studies [53], but higher than longitudinal studies that applied specific retention strategies trying to limit drop-outs [54, 55]. Cohorts characteristics seem to be a crucial factor in modulate retention rates, particularly depending by different study modalities employed. Indeed, highly educated women seem to prefer paper and pencil surveys, compared to on-line versions. However, at follow-up, paper and pencil modality showed lower retention rates compared to the on-line version [56]. These and results from other studies have to be taken into account for this and future on-line cohort studies to implement focused and specific retention strategies to limit drop-outs.

“One-year follow-up responders” were older, with higher educational level, and reported at the beginning better self-perceived cognitive and mental health, as well as more physical activity and vital plan. This is in line with what has been found in previous longitudinal studies that showed that low educational level, psychological distress and unhealthy life style factors, like physical inactivity, are associated to attrition [57, 58, 59]. This suggests that people with more interest in health maintenance and healthy lifestyles are those who tend to be more engaged in such studies, probably unavoidably introducing the observed population bias recruiting and bias follow-up in such studies.

### One year follow-up

When we compared the follow-up data with those acquired at baseline we found that over the course of a year participants slightly changed their habits, improving physical activity and sleep, but worsening their adherence to Mediterranean diet. However, these differences, even if statistically significant, were rather small in effect size. We hypothesize that those participants who already have healthy habits did not change them, considering especially that participants that completed the one-year follow-up showed better lifestyles at baseline. In order to test this we explored the relation between the one year change, as the difference between scores at one-year and at baseline, and the baseline characteristic of participants. Results revealed that participants with worst habits at the beginning are those who improved them the most, while those who already had good lifestyles remained stable or even worsened them. We explored the possibility that these results might have been driven by test-retest variability and a regression to the mean phenomenon [45, 60], rather than from real changes in participant’s habits. However, even after correcting for this [46] we still found a negative association between habits at the beginning of the study and improvement over a year, even though is clearly reduced in the magnitude.

These negative associations between habits at baseline and follow-up may be related to difficulties in maintaining good habits consistently over-time. This interpretation may have relevance for the implementation of multi-domain interventions aiming for simultaneous optimization of multiple lifestyles. Few studies to date have succeeded in such efforts and the present results may be important to consider in the setting of study design. However this interpretation should be taken with caution since as stated previously, the BBHI participants have continued access to ‘brain tips’ and ‘brain news’ through both the public and personalized dedicated web portals of the project. Therefore and beyond the fact that negative associations between baseline and follow up habit status may be explained by a ‘regression to the mean’ phenomena, we cannot determine if habits change from baseline to follow-up could rather reflect a ‘natural course’ or the effect of a ‘low level of intervention’, because a control group (i.e. participants assessed both at baseline and at follow up but not participating in the BBHI) was not included in our study.

### Endpoints analysis

The one year follow-up survey revealed that 73 participants had been diagnosed in the last year with one of the neurological or neuropsychiatric condition that represents an endpoint for the study. This represents slightly over 3% incidence of new diagnoses for the cohort of previously healthy 40–65 year olds, which is in line with the statistical estimation that we made based on Spanish available epidemiological data for these diseases (Fig 1, [1–10]). Moreover, in line with recent epidemiological reviews [61] and considering the age-range and the high proportion of women in the cohort, the most incident condition was major depression (76.7% of all reported new diagnoses; [1–10].

We explored the relation between lifestyles and participants characteristics and the incidence of a new diagnosis of depression. First, we looked at the association between habit changes over the year and diagnosis and we found that increased purpose in life represented a protective factor.

Purpose in life is a psychological construct that represent the notion of having a sense of goal and future oriented behavior that may confer more capacity to manage and tolerate changes and demanding temporal situations. Purpose in life is one of the six components of Carol D. Ryff’s prevalent model [62], along with autonomy, personal growth, environmental mastery, positive relationships, and self-acceptance. It has been previously related with biological risk factors such as inflammatory markers [63], cardiovascular diseases [64], and other health related outcomes [65]. Purpose in life has been also related with cognitive function in healthy adults, mortality, and to the risk of Alzheimer’s disease (AD) and mild cognitive impairment (MCI; [66, 67, 68, 69]).

Second we explored if certain baseline modifiable and non-modifiable condition may represent a risk or protective factor. Results revealed that women were at higher risk for diagnosis of depression (see Table 6), while higher levels of sleep quality and Sense of coherence represent protective factors to develop this condition. Considering that almost 80% of the diagnosis was depression these results are not surprising. There is consistent evidence in literature suggesting that women are twice as likely to develop depression that man [11, 12], and also sense of coherence and sleep have been previously associated with depression [70, 71]. The role of gender as a risk factor for depression has been related to the more stressful role of women in society and the resulting lower degrees of fulfillment, and the greater vulnerability to negative life events of friends and family given the increase sense of responsibility, copying style and biological response to stress [12, 72].

Sense of Coherence, is not a cultured-bound construct that allows individuals to identify strategies and resources available to manage stressors, and it has been related to a variety of health outcomes, including depression ([71, 73]; see [74] for a review). Sense of coherence is a core component in the salutogenic model of Aaron Antonovsky [75] and is defined as “a global orientation that expresses the extent to which one has a pervasive, enduring though dynamic feeling of confidence that (1) the stimuli deriving from one’s internal and external environments in the course of living are structured, predictable, and explicable; (2) the resources are available to one to meet the demands posed by these stimuli; and (3) these demands are challenges, worthy of investment and engagement’ [76]. Our findings are consistent with studies that argue that a high sense of coherence allows to better cope with stressors and represents a health promoting factor [71] associated with reduced mortality, increased well-being, and reduced incidence of depression [77, 78, 79].

Finally, the relation between sleep disturbance and mental health is well documented in literature, particularly with insomnia. This condition indeed has been consistently related with depression [80, 81, 82] and a recent meta-analysis showed that it is a very strong predictor of this condition [83]. When we looked at the relation between these modifiable and non-modifiable variables in regards to the risk of a new neurologic or psychiatric diagnosis we found that sleep quality partially mediated the relation between gender and risk of depression. The association between gender and specific sleep disorders is known and well documented in literature (see [84] for review): women are more likely than men to develop insomnia, restless leg syndrome and REM sleep disorders, while men are at double the risk of Obstructive Sleep Apnea than women. Given such findings, it is not surprising that part of the risk to develop depression is linked to poor sleep quality.

These findings stress the importance of promoting sleep quality in middle age people, particularly in women, and extend to the field of prevention previous results on the positive effect of sleep quality interventions in the treatment of mental health illnesses [85]. Future intervention studies should assess the impact of sleep quality promotion programs in reducing the incidence of mental health related pathology.

### Limitation and future directions

This study has some possible limitation/confounders. The on-line administration of the questionnaires, as well as representativeness of the cohort and statistical bias may limit the strong of our results.

Moreover 93.7% of participants are living in the metropolitan area of Barcelona and are born in Spain (and more than 99% in Europe), and represent a rather homogeneous Caucasian population. The fact that our participants may reflect culturally related of Catalan/Spanish population is a possible limitation in extend our results to other populations. Further studies in other cultural contexts and other regions of the world would be needed to address this issue.

On the other hand the big number of participants represents a positive factor and in this sense the retention challenge mentioned above represents a very crucial point for the future of the initiative. During this year participants will continue to respond periodically to new surveys and for the end of 2019 they will complete a new 1-year follow up for.

This three time points will allow us to exhaustively and in a more complete way confirm our present results and better describe the longitudinal changes in our cohort.

## Supporting information

S1 File All the data necessaries to replicate the analyses contained in the paper. (XLSX)
